# The Utility of Abdominal Ultrasound Following Negative Computed Tomography in Diagnosing Acute Pancreatitis

**DOI:** 10.7759/cureus.27752

**Published:** 2022-08-07

**Authors:** Chadley D Froes, Kiranpreet Gosal, Pratishtha Singh, Victor Collier

**Affiliations:** 1 Internal Medicine, Grand Strand Medical Center, Myrtle Beach, USA

**Keywords:** gallstone pancreatitis, comparing ct to us, ct (computed tomography) imaging, transabdominal ultrasound, acute pancreatitis

## Abstract

Aim

Acute pancreatitis is a diagnosis established by fulfillment of at least two out of three clinical features, including epigastric pain, elevated lipase, and/or radiographic evidence of acute pancreatitis. Computed tomography of the abdomen and pelvis (CTAP) is the gold standard imaging modality for evaluating acute pancreatitis. Although abdominal ultrasound (AUS) is increasingly utilized given the widespread availability and high sensitivity and specificity for detecting gallstone-related complications, including gallstone pancreatitis, the leading cause of acute pancreatitis in the US. However, recent literature has concluded that performing AUS following a negative CTAP rarely led to changes in management and imparted an increased length of service (LOS) in the ED. Our study investigated whether a similar relationship was observed when managing acute pancreatitis in the inpatient setting. We aimed to quantify how performing AUS influenced inpatient LOS for patients admitted for acute pancreatitis without radiographic evidence of acute pancreatitis on CTAP. We also aimed to quantify how AUS influenced the likelihood of subsequent intervention via endoscopic retrograde cholangiopancreatography (ERCP) or cholecystectomy, including the relative impact of certain demographic or clinical features.

Methods

A retrospective analysis was performed using a cohort of 6069 patient encounters extracted via the HCA Healthcare enterprise data warehouse (EDW) database. Inclusion criteria were all adult patients with an index admission for acute pancreatitis between January 1 and December 31, 2019, who underwent CTAP during admission. Patients younger than 18 years, with prior cholecystectomy, or without documentation of demographic or clinical data of interest were excluded. The primary outcome was to quantify how performing AUS within 48 hours impacted LOS for patients admitted for acute pancreatitis following negative CTAP. Secondary outcomes examined whether AUS changed management (i.e., per likelihood of subsequent ERCP or cholecystectomy). This included determining the influence of various demographic or clinical characteristics on the likelihood of intervention via ERCP or cholecystectomy. Linear regression was used to determine the effect of performing AUS on the duration of LOS. Logistic regression was used for covariate analysis based on demographic (BMI, sex, race, age) and clinical data (comorbid conditions, abnormal labs, and vital signs).

Results

Patients with acute pancreatitis who underwent AUS within 48 hours had a reduced LOS of 1.099 days. Patients who underwent AUS were 1.126 times more likely to undergo subsequent ERCP than those who received CTAP alone. Patients who received AUS following CTAP were also 2.711 times more likely to undergo subsequent cholecystectomy. Increasing age and BMI were correlated with an increased likelihood of ERCP and cholecystectomy. Males were less likely to undergo cholecystectomy (OR = 0.753) and ERCP (OR = 0.815) compared to females.

Conclusion

Performing AUS within 48 hours following negative CTAP in this cohort of patients admitted for acute pancreatitis was associated with a decreased LOS. Furthermore, patients who underwent AUS were more likely to undergo ERCP and even more likely to undergo cholecystectomy. The likelihood of ERCP and cholecystectomy increased proportionally to both age and BMI. Females were more likely than males to undergo subsequent ERCP or cholecystectomy.

## Introduction

As described by the Revised Atlanta classification, acute pancreatitis is diagnosed by the fulfillment of at least two out of three of the following clinical criteria: epigastric abdominal pain, elevated lipase level (more than three times the upper limit of normal), and/or radiographic evidence of pancreatitis [1]. Computer tomography of the abdomen and pelvis (CTAP) is frequently the imaging modality of choice for establishing the radiographic evidence needed for diagnosis. However, abdominal ultrasound (AUS) is typically also obtained to assess for biliary and peripancreatic pathology [2-4]. CTAP is classically the gold standard imaging modality, primarily since it may provide insight into the severity of acute pancreatitis, especially alongside evidence-based Ranson and APACHE-II risk stratification scoring [4,5]. In addition, AUS may offer some utility given the high sensitivity for diagnosing cholelithiasis and various gallstone pathologies at the bedside without additional radiation exposure. Both modalities offer a reasonably high degree of diagnostic efficacy and are routinely used in the work-up of acute pancreatitis. For example, CTAP is 60-88% sensitive and 97-100% specific for detecting choledocholithiasis, while ultrasound (US) has a sensitivity of 50-80% and a specificity of 90% [3,5]. However, US is known for its limitations with respect to operator skill and body habitus, the latter of which is primarily determined by patient characteristics such as age and BMI [6]. Moreover, in the first 48 hours of acute pancreatitis, US may have additional limitations imparted by the presence of paralytic ileus and overlying bowel gas, which obstruct or distort the field of view and therefore decreases the sensitivity of imaging while also subjecting the patient to discomfort and pain [3,7]. Despite these limitations, contrast-enhanced US is emerging as a highly sensitive and specific (>90%) modality for the detection of necrotizing foci or other complications while also having high sensitivity for determining the severity of acute pancreatitis [8]. Nonetheless, current literature fails to establish whether obtaining AUS leads to clinically meaningful changes in the management of acute pancreatitis following negative CTAP or whether clinical or demographic characteristics influence these changes. Prior work has specifically evaluated the utility of US after a negative CTAP in the setting of the ED. More specifically, one study followed 335 patients over a three-year follow-up period. It concluded that AUS was not only unlikely to change management in the setting of negative CTAP, but it was also associated with an increased length of service (LOS) in the ED and an increased out-of-pocket cost to the patient [9]. These conclusions raise questions regarding the utility of AUS as a core component of the standard diagnostic work-up of acute pancreatitis. Moreover, further investigation is necessary to substantiate whether their conclusions are generalizable to patients admitted to the general inpatient medical service, given that their patient population was specifically limited to the ED. It should be worth noting that performing AUS following negative CTAP represents a deviation in management from the standard of care for treating patients with acute pancreatitis. Most international guidelines recommend performing AUS on admission for all patients with acute pancreatitis followed by CTAP in those without identifiable cause on US [10-13].

Our study was designed to evaluate the utility of performing AUS in patients admitted for acute pancreatitis without radiographic evidence on CTAP. We specifically aimed to determine the degree to which performing AUS within 48 hours of admission for acute pancreatitis influenced LOS, including whether AUS changed the likelihood of subsequent cholecystectomy or endoscopic retrograde cholangiopancreatography (ERCP) when controlling for all other variables. This includes the identification of any pertinent clinical metrics (i.e., vital signs, lab values, BMI) or demographic characteristics (i.e., age, race, sex) that may be associated with an increased likelihood of ERCP and/or cholecystectomy.

## Materials and methods

Study design

We performed a retrospective study that evaluated patient information from hospitals in the Southeast region of the United States. Data were obtained from the HCA Healthcare Enterprise Data Warehouse (EDW) database, which includes inpatient laboratory and pharmacy claim codes with the International Classification of Diseases (ICD) Revision 10 throughout the period of index admission. The study was conducted in compliance with the HCA Healthcare requirements and received an Institutional Review Board (IRB) exempt determination through centralized algorithms for research rules on IRB exemptions.

Cohort

**Figure 1:**
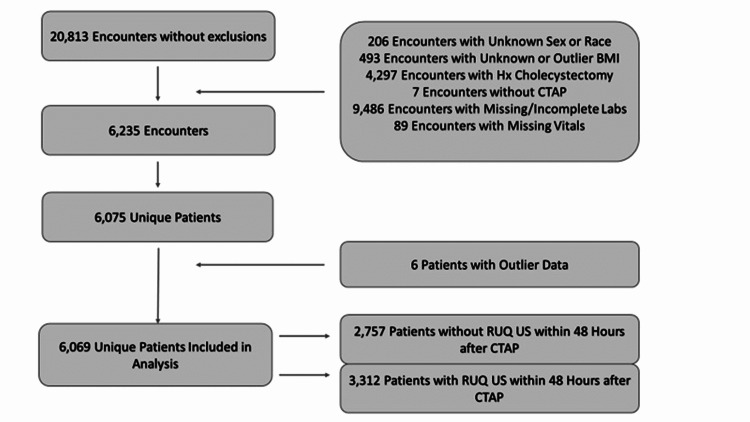
Summary of the exclusion process.

All adults aged 18 and older with an index admission during the calendar year of 2019 (i.e., January 1st through December 31st) with a diagnosis of acute pancreatitis per documented ICD10 code(s) were included in the study. Exclusion criteria were patients under 18 years of age, patients with prior cholecystectomy, or patients who did not undergo initial imaging via CTAP. A summary of the exclusion process is depicted in Figure 1. The study included a total of 6069 unique patient encounters. The study index date was defined as the date of hospitalization, with index admission dates ranging from January 1, 2019, to December 31, 2019, or the 2019 calendar year.

Outcomes and exposure coding

The primary outcome was to determine the influence of performing AUS within 24 hours on the duration of LOS (measured in days). Secondary outcomes examined the likelihood of patients undergoing subsequent ERCP or cholecystectomy when controlling for all other variables. Further covariate analyses were also performed to determine the effect of other clinical and demographic characteristics on the likelihood of intervention for patients who underwent cholecystectomy or ERCP.

Covariates

Clinical metrics and demographic characteristics were extracted for each patient throughout the duration of index admission and recorded in the medical record. In addition, pertinent data were extracted retrospectively for all patient encounters that met exclusion criteria (Figure 1). Demographic characteristics included sex, race, and age. Clinical data points examined included any pertinent lab values outside of accepted reference ranges out of those routinely performed for acute pancreatitis (e.g., complete blood count, lipase, comprehensive metabolic panel), BMI, and vital signs, including heart rate (HR), respiratory rate (RR), temperature, mean arterial pressure (MAP), and oxygen saturation.

Statistical analysis

Linear regression analysis was performed to compare LOS in patients who underwent AUS within 48 hours to those who did not undergo AUS, assuming all other variables are held constant. The linear regression coefficient was used to compare LOS using an alpha value of 0.05 as the threshold for statistical significance. Binary logistic regression with Fisher's optimization was implemented to determine whether performing AUS within 48 hours of admission significantly impacted the likelihood of a patient receiving subsequent interventions, specifically ERCP and cholecystectomy, when controlling for age, sex, BMI, lab values, and vital signs. This included clinical metrics such as abnormal lab values, vital signs (HR, RR, MAP, temperature), and a diagnosis of cholecystitis. Note that separate logistic regression analyses were performed for ERCP and cholecystectomy, respectively, using a p-value ≤0.05 as the threshold for statistical significance.

## Results

Descriptive statistics

A total of 6,069 unique patient encounters were included in the study after meeting the criteria described earlier. Of these, 54.6% (n = 3,312) underwent AUS within 48 hours of admission compared to 45.4% (n =2,757) who did not. Both groups were similarly distributed with respect to sex and race, with both groups exhibiting a predominantly white racial demographic of approximately 65%. Of those who did not undergo AUS within 48 hours, 18.20% (n = 503) underwent AUS at a later time (i.e., greater than 48 hours into admission). An overview of the number and percentage of patients with various clinical characteristics is summarized in Table 1.

**Table 1:** Descriptive statistics of study population split based on whether or not AUS was performed within 48 hours. AUS: Abdominal ultrasound; ERCP: Endoscopic retrograde cholangiopancreatography.

Characteristics		No AUS performed		AUS within 48 hours	
		n	%	n	%
Sex	Female	1,199	43.50%	1,554	46.90%
	Male	1,558	56.50%	1,758	53.10%
Race	Other	950	34.50%	1,135	34.30%
	White	1,807	65.50%	2,177	65.70%
AUS anytime during admission	No	2,254	81.80%	0	0.00%
	Yes	503	18.20%	3,312	100.00%
Diagnosis of cholecystitis	No	2,333	84.60%	2,199	66.40%
	Yes	424	15.40%	1,113	33.60%
ERCP	No	2,574	93.40%	2,862	86.40%
	Yes	183	6.60%	450	13.60%
Cholecystectomy	No	2,507	90.90%	2,478	74.80%
	Yes	250	9.10%	834	25.20%

Descriptive statistical characteristics such as mean, median, range, and SD were calculated for various clinical characteristics in order to compare average values between the two groups (no AUS and AUS within 48 hours). Most notably, patients who underwent AUS within 48 hours had a mean LOS duration of 4.897 days (SD = 6.028 days), compared to 6.163 days (SD = 9.417 days ) in those who did not undergo AUS. This suggests an average reduction in LOS in those who underwent AUS. Most of the other included characteristics had similar mean values when comparing the two groups, as described in Table 2 below.

**Table 2:** Comparison of various clinical characteristics based on whether or not AUS was performed within 48 hours. * = excluded from final analysis due to multicolinearity. AUS: Abdominal ultrasound; ALT: Alanine transaminase; AST: Aspartate aminotransferase; BUN: Blood urea nitrogen.

Clinical characteristics	No AUS performed					AUS within 48 hours				
	Mean	Median	Minimum	Maximum	Standard Deviation	Mean	Median	Minimum	Maximum	Standard Deviation
Age	53.566	54	18	90	17.225	54.137	55	18	90	18.009
Length of Stay (Days)	6.163	3	0	133	9.417	4.897	3	0	99	6.028
BMI	29.626	28.659	11.961	73.509	7.364	30.651	29.464	13.063	76.303	7.709
ALT (100 units/L)	0.689	0.315	0.06	26.7	1.392	1.002	0.408	0.057	33.648	1.739
AST (100 units/L)	0.723	0.297	0.04	45.07	2.064	1.012	0.377	0.01	167.925	4.3
BUN (mg/dL)	17.673	13.438	2	118	14.023	16.007	12.75	1.4	169.667	12.105
Creatinine (mg/dL)	1.282	0.88	0.221	21.133	1.485	1.114	0.85	0.2	14.659	1.074
Lipase (100 units/L)	22.81	9.057	0.1	361.915	37.847	33.301	14.387	0.1	494.71	46.025
Triglycerides (100 mg/dL)	2.881	1.28	0.02	116.49	5.884	2.388	1.12	0.14	108.18	5.258
Mean Arterial Pressure	95.183	94.526	61.192	143.111	11.111	95.276	94.821	61.74	132.643	10.299
Systolic Pressure*	133.144	132.167	91.731	206.667	17.102	133.699	132.59	88.079	188.071	16.778
Diastolic Pressure*	76.202	75.568	44	113.833	9.71	76.065	75.398	43.535	109.433	8.766
Pulse	82.766	81.977	45.778	138.352	13.988	81.019	79.226	43.774	135.401	14.165
Respiratory Rate	17.959	17.314	12	47.562	2.595	17.617	17.23	13	38.225	2.185
SPO2%*	96.396	96.533	87.232	100	1.718	96.248	96.357	86.48	100	1.733

Length of service

The primary outcome was to determine how performing AUS within 48 hours impacted the duration of LOS in patients admitted for acute pancreatitis compared to control (i.e., no AUS performed). Linear regression coefficient comparing those who underwent AUS within 48 hours to LOS revealed a statistically significant expected reduction in LOS by 1.099 days when controlling for all other variables (p < 0.001, alpha = 0.05, 95% CI = 0.737-1.460 days), as shown in Table 2. The mean LOS for patients who underwent AUS within 48 hours was 5.472 days (SD: 9.417 days) compared to a mean LOS of 9.417 days (SD: 6.028 days) in patients who did not undergo AUS within 48 hours. Patients who receive AUS within the first two days of admission are therefore expected to have a shorter LOS. The diagnosis of cholecystitis was noted for significantly greater expected LOS of 2.478 days (p < 0.001, alpha = 0.05, 95% CI 2.046-2.909 days). Therefore with a diagnosis of cholecystitis tend to have longer hospitalizations than others who do not. Of clinical metrics included in our analysis, such as MAP, HR, and RR were associated with a mild but statistically significant increase in LOS, while an inverse relationship between MAP and LOS. Elevated HR, temperature, and RR were also correlated with increased LOS. Specific expected changes to LOS are noted in Table 3.

**Table 3:** Linear regression analysis comparing multiple independent clinical variables to duration of length of service (dependent variable). AUS: Abdominal ultrasound; ALT: Alanine transaminase; BUN: Blood urea nitrogen.

Model	Unstandardized coefficients		Standardized coefficients	t	Significance	95% CI	
								B	Std. Error	Beta	Lower Limit	Upper Limit
	(Constant)	-59.427	12.513			N/A	-4.749	<0.001	-83.956	-34.898
AUS within 48 hours	-1.099	0.185	-0.07	-5.953	<0.001	-1.46	-0.737
Age	0.008	0.006	0.019	1.44	0.15	-0.003	0.019
Sex	0.05	0.186	0.003	0.268	0.789	-0.314	0.414
BMI	-0.005	0.012	-0.005	-0.406	0.685	-0.029	0.019
ALT (100 Units)	0.011	0.058	0.002	0.185	0.853	-0.103	0.125
BUN (1 Unit)	0.125	0.01	0.209	13.02	<0.001	0.106	0.144
Creatinine	-0.157	0.089	-0.026	-1.762	0.078	-0.331	0.018
Lipase (100 Units)	-0.008	0.002	-0.042	-3.601	<0.001	-0.012	-0.004
Triglycerides (100 units)	-0.074	0.017	-0.053	-4.39	<0.001	-0.108	-0.041
Mean arterial pressure	-0.067	0.009	-0.092	-7.462	<0.001	-0.085	-0.049
Pulse	0.092	0.007	0.167	12.668	<0.001	0.078	0.106
Respiratory rate	0.644	0.043	0.197	14.971	<0.001	0.559	0.728
Temperature (F)	0.514	0.128	0.047	4.024	<0.001	0.263	0.764
Cholecystitis	2.478	0.22	0.139	11.247	<0.001	2.046	2.909

ERCP

Logistic regression analysis was performed to determine the likelihood of a patient undergoing ERCP relative to various clinical and demographic parameters. Given a p-value of 0.260, there is inadequate statistical significance to state that undergoing AUS within 24 hours increases the likelihood of a patient undergoing ERCP (p = 0.260, OR = 1.126, 95% CI [0.916, 1.385]). There may be clinical significance, as patients undergoing AUS within 48 hours following negative CTAP are 1.126 times more likely to undergo ERCP. Meanwhile, patients with an elevated BMI (p = 0.026, OR = 1.014, 95% CI [1.002, 1.027]), elevated ALT levels (p <0.001, OR = 1.113, 95% CI [1.062, 1.166]), elevated lipase levels (p = 0.003, OR = 1.003, 95% CI [1.001, 1.005]), and a diagnosis of cholecystitis (p < 0.001, OR = 15.720, 95% CI [12.534, 19.715]) are more likely to undergo ERCP than patients with a lower BMI, lower ALT levels, lower lipase levels, or patients without a diagnosis of cholecystitis (Table 4). Moreover, each one-year increase in age was also associated with a statistically significant increased odds of undergoing ERCP (p = 0.002, OR = 1.009, 95% CI [1.003 and 1.015]). When comparing males to females, the difference in log-odds for ERCP is expected to be -0.204 units, assuming all other variables are constant. Using sex as a predictor variable, male patients are therefore 0.815 times as likely (less likely) to have an ERCP than female patients (p = 0.042, OR = 0.815, 95% CI [0.670, 0.993]). There is, therefore, sufficient evidence to state that male patients are less likely to require an ERCP than female patients. 

**Table 4:** Logistic regression analysis comparing the effect of various clinical parameters on the likelihood of undergoing ERCP. Exp (B) = odds ratio (OR) d.f.: degrees of freedom; AUS: Abdominal ultrasound; ALT: Alanine transaminase; ERCP: Endoscopic retrograde cholangiopancreatography.

Clinical parameter	B	Standard Error	Wald	d.f.	Significance	Exp(B)	95% CI	
							Lower Limit	Upper Limit
AUS within 48 hours	0.119	0.106	1.269	1	0.26	1.126	0.916	1.385
Age	0.009	0.003	9.486	1	0.002	1.009	1.003	1.015
Sex	-0.204	0.101	4.121	1	0.042	0.815	0.67	0.993
BMI	0.014	0.006	4.927	1	0.026	1.014	1.002	1.027
ALT (100 Units)	0.107	0.024	19.961	1	< .001	1.113	1.062	1.166
BUN	-0.003	0.006	0.324	1	0.569	0.997	0.985	1.008
Creatinine	-0.112	0.072	2.397	1	0.122	0.894	0.777	1.03
Lipase (100 units)	0.003	0.001	8.639	1	0.003	1.003	1.001	1.005
Triglyceride (100 units)	-0.014	0.016	0.776	1	0.378	0.986	0.956	1.017
Mean Arterial Pressure	-0.009	0.005	3.231	1	0.072	0.991	0.98	1.001
Pulse	-0.006	0.004	1.987	1	0.159	0.994	0.986	1.002
Respiratory Rate	-0.008	0.025	0.108	1	0.742	0.992	0.944	1.042
Temperature (F)	0.08	0.072	1.236	1	0.266	1.083	0.941	1.246
Cholecystitis	2.755	0.116	568.475	1	< .001	15.72	12.534	19.715
Constant	-10.805	7.016	2.372	1	0.124	0	N/A	N/A

Cholecystectomy

A separate logistic regression analysis was performed to determine the influence of the same clinical and demographic variables on the likelihood of subsequent cholecystectomy. In this case, AUS within 24 hours was significantly associated with an increased likelihood of undergoing cholecystectomy (p < 0.001, OR = 2.711, 95% CI [2.312, 3.179]), such that these patients are 2.711 times more likely to proceed to cholecystectomy. Not unlike ERCP, older patients (p < 0.001 , OR = 1.014, 95% CI [1.010, 1.019]), patients with a higher BMI (p < 0.001, OR = 1.036, 95% CI [1.027, 1.046]), higher ALT levels (per 100 units; p < 0.001, OR = 1.318, 95% CI [1.261, 1.378]), and higher k=lipase levels (per 100 units: p < 0.001, OR = 1.005, 95% CI [1.003, 1.006]) are more likely to undergo a cholecystectomy than younger patients, patients with a lower BMI, lower ALT levels, and lower lipase levels. Similar relationships were also found in sex, such that male patients are 0.753 times as likely (i.e. less likely) to undergo a cholecystectomy compared to female patients (p < 0.001, OR = 0.753, 95% CI [0.648, 0.873]). These and other statistics are summarized in Table 5.

**Table 5:** Logistic regression analysis comparing the effect of various clinical parameters on likelihood of undergoing cholecystectomy. Exp(B) = odds ratio (OR) d.f. = degrees of freedom; AUS: Abdominal ultrasound; ALT: Alanine transaminase; BUN: Blood urea nitrogen.

Clinical parameter	B	Standard Error	Wald	d.f.	Significance	Exp(B)	95% CI	
							Lower Limit	Upper Limit
AUS within 48 hours	0.997	0.081	150.368	1	< .001	2.711	2.312	3.179
Age	0.014	0.002	37.083	1	< .001	1.014	1.01	1.019
Sex	-0.284	0.076	13.98	1	< .001	0.753	0.648	0.873
BMI	0.035	0.005	57.53	1	< .001	1.036	1.027	1.046
ALT (100 Units)	0.276	0.023	150.363	1	< .001	1.318	1.261	1.378
BUN	-0.034	0.006	33.365	1	< .001	0.967	0.956	0.978
Creatinine	-0.012	0.054	0.05	1	0.824	0.988	0.889	1.098
Lipase (100 Units)	0.005	0.001	43.739	1	< .001	1.005	1.003	1.006
Triglycerides (100 Units)	-0.106	0.02	28.419	1	< .001	0.9	0.865	0.935
Mean Arterial Pressure	-0.008	0.004	4.621	1	0.032	0.992	0.984	0.999
Pulse	-0.004	0.003	1.57	1	0.21	0.996	0.99	1.002
Respiratory Rate	-0.082	0.022	13.319	1	< .001	0.922	0.882	0.963
Temperature (F)	0.065	0.054	1.456	1	0.228	1.067	0.96	1.187
Constant	-7.477	5.305	1.987	1	0.159	0.001	N/A	N/A

## Discussion

Gallstone pancreatitis accounts for approximately 40-70% of all cases of acute pancreatitis in the developed world, representing at least two-thirds of all cases when combined with alcohol abuse [1,3]. Given its prevalence in the pathophysiology of acute pancreatitis, detection of gallstone disease is of relative importance in the initial diagnostic evaluation of acute pancreatitis, especially given that radiographic evidence is often needed to fulfill clinical criteria to make the diagnosis. As discussed above, CTAP has an extremely high diagnostic yield, especially when evaluating disease severity or various complications of acute pancreatitis, such as necrotic foci. On the other hand, AUS may offer superior sensitivity to CTAP in detecting gallstone-associated causes of acute pancreatitis and can be performed at the bedside without exposure to ionizing radiation [3,4]. However, AUS has known limitations that decrease sensitivity depending on factors such as operator technique, body habitus, bowel gas or ileus presence, or age-related anatomic variability [2,5,6]. 

From a clinical perspective, the combined synergistic role of US and CT in diagnosing acute pancreatitis is supported by the inclusion of both modalities in multiple evidence-based guidelines. Specifically, high-quality evidence supports performing an ultrasound on admission to evaluate for biliary causes of acute pancreatitis, followed by CT imaging in cases with uncertain ultrasound findings or severe clinical manifestations [9-12]. However, the role of AUS in the context of negative CTAP findings is less well defined by most guidelines. Recent literature has also concluded that performing AUS within 48 hours following negative CTAP rarely led to changes in management and may even increase the LOS for patients who presented to the ED with acute pancreatitis [9].

This was the focus of our study's primary outcome, the results of which challenge prior conclusions and instead suggest that performing AUS within 48 hours of negative CTAP imparts a reduced LOS. This discrepancy may be related to the context of the patient population in question, which was addressed in the study in question [9]. More specifically, the previous study consisted of ED patients from a single-center database, whereas our study utilized data from inpatient admissions across multiple hospitals affiliated with HCA Healthcare. Moreover, since the previous study utilized a secondary cohort of patients with negative CTAP only to determine LOS, this may have introduced false negatives that contributed to increased LOS. Finally, the seemingly contradictory decreased LOS observed in our study may reflect a decreased time to diagnosis following negative CT imaging. Rapid diagnosis facilitated more prompt intervention, decreasing inpatient LOS. The data regarding our secondary outcomes may also support the notion that US may increase the likelihood of prompt intervention. More specifically, our results demonstrated that performing AUS following negative CTAP increased the likelihood of subsequent intervention via cholecystectomy or ERCP. This discrepancy could be explained by the presence of false negatives on initial CTAP, which could be picked up on follow-up US and, therefore, prompt intervention via ERCP or cholecystectomy. Alternatively, the contrasting results of our primary and secondary outcomes may suggest that patient selection is an essential component of the usefulness of AUS following negative CTAP. AUS may indeed have more favorable utility in the inpatient setting, with decreased diagnostic or prognostic yield in the ED. 

The impact of age and BMI in our results are less easily explained, especially since it is well established that US becomes less efficacious as BMI increases or with the higher prevalence of anatomic variability observed in older patients [4,6]. In this sense, one might expect AUS to have less of an impact on management as BMI or increasing age since diagnostic accuracy decreases as a function of both variables. However, the observed increased utilization of ERCP and cholecystectomy in our study with increasing age and BMI may be due to a greater degree of diagnostic uncertainty, such that providers have a lower threshold for intervention in patients with less reliable imaging. Unfortunately, the presence or absence of diagnostic uncertainty is difficult, if not impossible, to measure, especially in retrospective studies that often lack standardization of documentation. Alternatively, providers may be more inclined to pursue definitive operative intervention in patients with a higher likelihood of clinical decompensation, such as those with elevated BMI or advanced age. Despite an exhaustive review of current academic literature, an explanation for why an increased BMI may impart a greater likelihood of ERCP has not been well established in current academic literature. However, obesity has been established as an independent risk factor for developing post-ERCP pancreatitis, potentially due to a low-grade chronic inflammatory state related to a decreased level of adiponectin, an anti-inflammatory adipokine [14]. Nonetheless, it is reasonable to conclude that patient selection influences the utility of AUS following negative CTAP and may have a greater effect on management in patients with elevated BMI or advanced age. These relationships could be further delineated as the focus of future prospective or retrospective studies.

Limitations

As a retrospective study, it is essential to interpret observational data with caution as findings may have been influenced by unmeasured or unidentified confounders, including potential variability in collection or documentation by providers. In addition, the potentially arbitrary designation of ICD10 codes may also confound the results of retrospective data. Limitations of retrospective research, including the inability to control the homogeneity of collected and recorded data, are well established in the academic literature and the main caveat of retrospective research [15]. Our study was also limited, given the inability to include management changes that occurred during outpatient follow-up. This is primarily due to the relative lack of uniformity in electronic medical record systems used by outpatient clinics, some of which may be outside of the HCA Healthcare medical system, which precludes inclusion in the EDW database. The relative likelihood of intervention is therefore restricted to interventions that occur during the period of index admission. Our results may therefore underestimate actual numbers since our data does not include the number of patients who underwent ERCP or cholecystectomy during outpatient follow-up after discharge.

## Conclusions

Our study focused on acute gallstone pancreatitis and the difference in LOS or likelihood of inpatient interventions (ERCP or cholecystectomy) when patients underwent AUS after 48 hours of negative CTAP. Patients who underwent AUS had a significantly shorter LOS overall than those who did not. This may be due to a decreased time to diagnosis and subsequent intervention. Secondary outcomes evaluated the impact of AUS on inpatient management based on the likelihood of a patient undergoing ERCP or cholecystectomy. Patients were more likely to undergo ERCP if AUS was performed within 48 hours following a negative CTAP. However, this relationship was not statistically significant but may confer clinical significance. Patients were significantly more likely to undergo cholecystectomy when AUS was performed within 48 hours following negative CTAP. When controlled for other variables, the likelihood of undergoing ERCP or cholecystectomy also increases as a function of increasing age and BMI. Interestingly, the relationship between BMI and increased likelihood of ERCP is not well established in our review of the current literature, which focuses more on the increased likelihood for obese patients to develop acute pancreatitis following ERCP (i.e., post-ERCP pancreatitis). More large-scale prospective or retrospective studies would help clarify precisely why increased BMI is associated with an increased likelihood of undergoing ERCP in the setting of acute pancreatitis. In addition, AUS may be associated with an increased likelihood of intervention via ERCP/cholecystectomy due to a greater degree of diagnostic accuracy. Similarly, AUS may reduce LOS by reducing the time to diagnosis and subsequent definitive intervention via ERCP or cholecystectomy. Finally, patient selection may play an essential role in determining the relative utility of US following a negative CTAP, especially with increasing age or BMI.
